# Tracing Neanderthal ancestry patterns through successive population expansions in Europe

**DOI:** 10.1126/sciadv.aeg6161

**Published:** 2026-11-11

**Authors:** Alexandros Tsoupas, Claudio S. Quilodrán, Jérémy Rio, Mathias Currat

**Affiliations:** ^1^ Department of Genetics and Evolution, University of Geneva, Geneva, Switzerland.; ^2^ Department of Evolutionary Biology and Environmental Studies, University of Zurich, Zurich, Switzerland.; ^3^ Institute of Genetics and Genomics in Geneva (IGE3), University of Geneva, Geneva, Switzerland.

## Abstract

During their expansion from Africa, modern humans interbred with Neanderthals, resulting in introgression of Neanderthal DNA into their genomes. This process contributed to a Southeast-to-Northwest gradient of Neanderthal ancestry across Europe. We investigate the population dynamics underlying the emergence and persistence of this gradient through two successive human range expansions. We developed a three-population, layered, spatially explicit simulation framework to track the spatiotemporal evolution of Neanderthal ancestry under a model of interactions among Neanderthals, pre-Neolithic hunter-gatherers, and Neolithic farmers. Our results indicate that the ancestry cline emerged from the interplay between the direction of the initial hunter-gatherer expansion, the northern limit of the Neanderthal range, and strong reproductive isolation, without requiring natural selection. The similar direction of the subsequent Neolithic expansion explains the cline’s persistence and Neanderthal ancestry differences between hunter-gatherers and farmers. These findings demonstrate how spatiotemporal patterns in ancient DNA provide insights into past population dynamics.

## INTRODUCTION

Modern humans (*Homo sapiens*) originated in Africa (*1*) and expanded into Eurasia via the Middle-East (*2*) between ∼60,000 and 40,000 years before present (YBP). During this major dispersal, they encountered Neanderthals, a related lineage that diverged from modern humans approximately 520,000 to 630,000 years ago (*3*). Neanderthals occupied a broad geographic range across Eurasia, from the Iberian Peninsula (*4*) to the Altai Mountains (*5*), until their extinction ∼40,000 YBP (*6*), following several millennia of coexistence with modern humans (*6*, *7*). The sequencing of the Neanderthal genome has showed that hybridization occurred between the two lineages during this period, with modern human populations outside sub-Saharan Africa still carrying Neanderthal-derived DNA segments today (*8*).

On average, contemporary non-African modern human populations harbor ∼2% Neanderthal ancestry in their genomes (*3*), with individual levels ranging between 1 and 4% (*9*). It is assumed that after an initial rapid decline due to purifying selection (*10*, *11*), only a limited number of genomic regions were subject to selection due to their association with specific traits or diseases [e.g., (*3*, *12*–*14*)]. In contrast, the long-term stability of Neanderthal ancestry levels in Eurasian populations over the past 40,000 years suggests that most introgressed DNA persisted under neutral evolution (*9*), reflecting the predominance of demographic rather than selective processes. Further support for a demographic explanation comes from the observation that Neanderthal ancestry in Eurasian populations exhibits a spatial gradient that increases with distance from the Near East, consistent with a model in which limited admixture occurred between a resident population at demographic equilibrium and an expanding modern human population (*15*). Spatially explicit simulations predict that such interactions generate a gradient of introgressed alleles along the expansion axis (*16*). Such introgression gradients can arise and persist due to demographic and migratory processes alone, without invoking selection as a driving factor (*17*). Nevertheless, the consequences of multiple overlapping population expansions involving admixture remain largely unexplored.

It has recently been suggested that the cline of Neanderthal ancestry was established during the initial settlement of Europe by early modern humans and has persisted to the present (*15*). However, the long-term persistence of such a cline is unexpected given that successive episodes of migration, population partial turnover with admixture have profoundly reshaped the genomic composition of European populations throughout Prehistory [e.g., (*18*–*20*)]. In particular, a major genomic change occurred with the emergence of agriculture in the Near East, which began spreading into Europe around 8500 YBP and progressively replaced the hunter-gatherer (HG) lifestyle (*20*). This process, known as the Neolithic transition, was driven by the migration of early farming populations from Western Anatolia and the Aegean basin (*19*) into Europe (*21*, *22*). As these farming groups dispersed across the continent, they increasingly admixed with local pre-Neolithic HG populations (*23*), resulting in genomes that combined both Neolithic farmer (NF) and HG ancestries (*24*), with marked regional variation (*25*). It has been shown that the average level of Neanderthal ancestry in European populations declined during the Neolithic transition, driven by the arrival of early farmers who carried slightly lower levels of Neanderthal ancestry than HG (*15*). Counterintuitively, the spatial gradient observed in HG persisted in the post-Neolithic populations, although the overall amount of Neanderthal ancestry declined. The demographic processes responsible for reducing the amount of ancestry while maintaining the gradient direction remain unexplored.

Here, we investigated the population dynamic processes underlying the creation and persistence of spatiotemporal patterns of Neanderthal ancestry in two ancient European populations: the pre-Neolithic HG and the subsequent NF populations. To reproduce the gradient of Neanderthal ancestry observed in HG and its evolution following the Neolithic transition, we developed an original three-population, spatially explicit simulation framework, based on the program SPLATCHE3 (*26*). This approach enabled us to explore how Neanderthal ancestry developed across Europe during two successive population expansions with admixture, each characterized by distinct demographic, migratory, and interaction processes. We explored which combinations of demographic parameters could generate the spatiotemporal pattern of Neanderthal ancestry observed in European populations. For this, we used approximate Bayesian computation (ABC) (*27*) to identify the demographic scenarios and parameter values that best matched Neanderthal introgression estimates obtained from a published paleogenomic dataset of 715 European pre-Neolithic HG and NF. By formally comparing simulated and empirical summary statistics, ABC bypasses the need to compute the model likelihood when it is intractable due to its complexity. This simulation framework enabled a direct investigation of the spatial and temporal patterns of Neanderthal ancestry, along with a comparison of population dynamics between these two key episodes of population turnover involving admixture.

## RESULTS AND DISCUSSION

Our analysis comprised three consecutive steps (Fig. 1). First, we simulated Neanderthals and HG to test whether the observed Neanderthal ancestry pattern in HG was shaped by the northern extent of Neanderthal occupation. We simulated four scenarios in which Neanderthals expanded to different maximum latitudes (50°, 55°, 60°, and 65°N) and used ABC to identify the scenario that best matched the empirical data. Second, using the best-supported Neanderthal range, we added a farmer population layer and performed additional simulations to test whether the observed ancestry pattern depended on the directions of the successive HG and farmer expansions. We compared four scenarios in which either the HG or farmer expansion followed alternative main directions. Last, we used ABC under the best-supported scenario to infer key demographic parameters while simulating all three populations (Neanderthals, HG, and farmers).

**Fig. 1.**
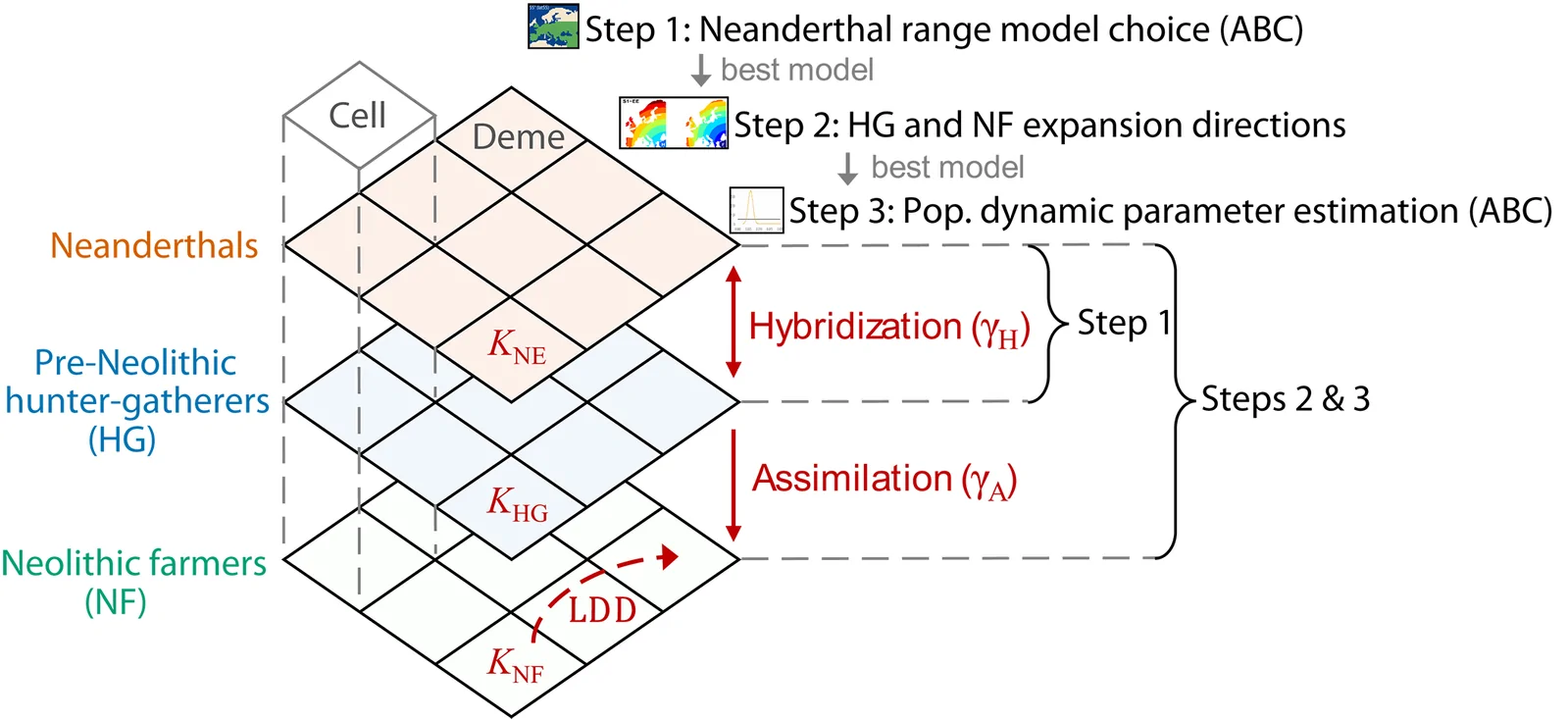
Schematic representation of the three-population, spatially explicit simulation framework and its three main inference steps. ABC stands for approximate Bayesian computation; estimated parameters: *Ki*, effective population size in population *i*; γ_H_, hybridization rate; γ_A_, assimilation rate. Each deme has its own demographic characteristics, and gene flow occurs between demes within the same cell geographic. Individuals can migrate to neighboring demes, while NF individuals can also disperse to more distant demes via LDD.

### Modern human arrival shaped the Neanderthal ancestry gradient

The large paleogenomic dataset, against which we compared the results of our simulations, is made up of 102 pre-Neolithic HG and 613 NF (Fig. 2), retrieved from the Allen Ancient DNA Resource (AADR) database (*28*). They shared, on average, 2.14% (SD = 0.38%) of their genome with Neanderthals. More specifically, pre-Neolithic HG have a mean proportion of Neanderthal ancestry estimated to 2.42% (SD = 0.42%) and NF to 2.08% (SD = 0.34%), the latter being statistically significantly lower than the former (two-sample *t* test *P* value = 6.2 × 10^–11^). Neanderthal ancestry in HG displays a statistically significant gradient in populations, increasing from the Southeast to the Northwest of the continent (Table 1). Overall, these patterns confirm the results of a previous analysis of the full AADR Eurasian dataset (*15*).

**Fig. 2.**
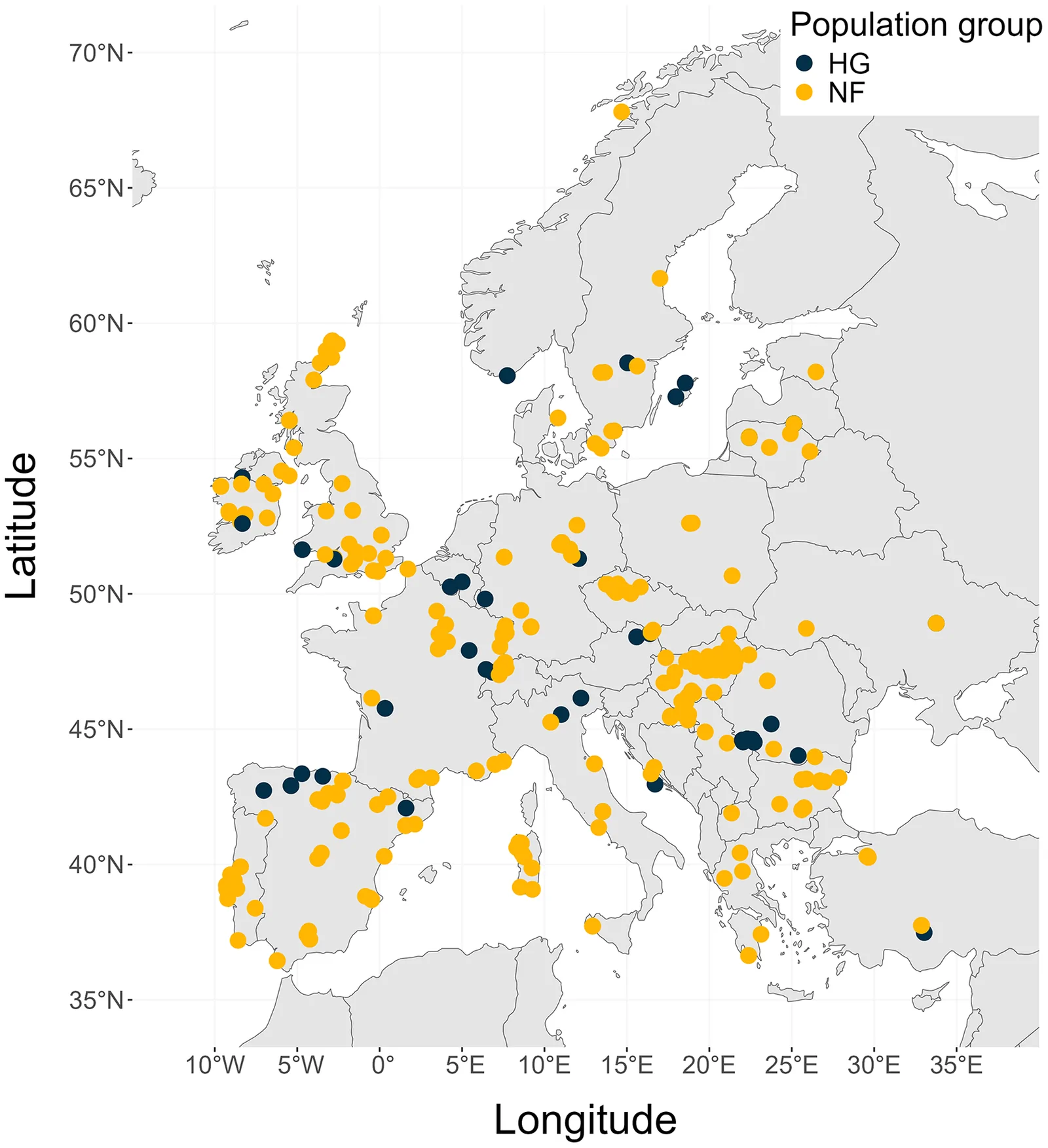
Geographic locations of the 715 individuals included in the analyses. Point colors indicate population group: red for pre-Neolithic HG (*n* = 102) and green for NF (*n* = 613). Overlapping points occur where sampling locations are in close proximity.

**Table 1.** Statistical models describing Neanderthal ancestry levels in European Paleolithic HG and NF. The coefficients of the linear models (in bold) were used as summary statistics for the ABC estimations. Marginal (*R*^2^_m_) and conditional (*R*^2^_c_) coefficients of determination indicate the variance explained by fixed effects alone and by both fixed and random effects, respectively. The response variable was log-transformed to ensure normally distributed residuals. Formula of the HG model called in the lm() function in R: log(α_f4_) ∼ distance + age. Formula of the NF model called in the lme() function of the nlme package in R: log(α_f4_) ∼ distance + age, random = ∼period | period, correlation = corLin(form = ∼distance, nugget = F).

Model	Variable	Coefficient	SE	*P* value	*R* ^2^
A) HG	Intercept	**−3.235**	0.155	<0.0001	*R*^2^_m_ = 0.198
(*n* = 90)	Distance	**5.31 × 10** ^**−05**^	2.19 × 10^−05^	0.0175	
	Age	**−2.71 × 10** ^**−04**^	6.19 × 10^−05^	<0.0001	
B) NF	Intercept	**−5.79**	0.786	<0.0001	*R*^2^_m_ = 0.075
(*n* = 424)	Distance	**2.4 × 10** ^**−05**^	1.15 × 10^−05^	0.0376	*R*^2^_c_ = 0.109
	Age	**7.26 × 10** ^**−04**^	3.14 × 10^−04^	0.0212	

A gradient of Neanderthal introgression is expected under a model of modern human range expansion with admixture (*29*). To assess whether the orientation of the observed gradient reflects the distribution of Neanderthals and the putative source of modern human expansion into Europe, we simulated four scenarios of HG expansion with a northern limit of the Neanderthal range varying between 50°N and 65°N (fig. S1). This range reflects the most plausible maximum latitude of Neanderthals in Europe (*30*, *31*), while their southern range extended until the Mediterranean and the Levant (*6*, *32*). We then performed model choice against the observed Neanderthal introgression gradient in HG using an ABC framework (see Materials and Methods for details). Note that this first series of simulations did not include a layer of farmer populations. All four investigated scenarios were able to reproduce, to some extent, the observed spatiotemporal gradient of Neanderthal ancestry in HG, as indicated by marginal density *P* values > 0.05 (table S1). The observed gradient is most likely explained by the scenario lat55 for which the maximum range is located at latitude 55°N, with a posterior probability of 41% (table S2). However, the cross-validation (fig. S2) indicates that the only model with good discriminatory power is lat50, whereas substantial overlap exists among lat55, lat60, and lat65 scenarios, in accordance with the decreasing amount of data available at higher latitudes. Our analysis thus favors a northern Neanderthal range limit above 50°N but is not strongly able to identify the exact location of this limit. Overall, our simulations suggest that the observed spatiotemporal pattern of Neanderthal ancestry in HG is best reproduced with a northern Neanderthal range limit at 55°N, whereas higher limits are increasingly unlikely to generate the observed gradient orientation (table S3). The fact that the lat50 model is the second best-supported model (table S2) and reproduces the spatial and temporal gradients almost as well as the lat55 model (table S3) suggests that the optimal northern Neanderthal range limit may lie between these two latitudes. However, the resolution of our simulations does not allow us to estimate this limit more precisely.

Notably, this estimate of ∼55°, derived solely from paleogenomic data, corroborates archaeological and chronological evidence, which place the northernmost extent of Neanderthal presence at a similar latitude [(*31*) and references therein], as well as habitat suitability models which place it at the same latitude or slightly farther south at ∼50° latitude (*33*). Although sporadic or low-density occupations may have occurred farther north, for example, in parts of southern Scandinavia, which appear environmentally suitable for Neanderthal habitation (*30*), no definitive evidence of Neanderthal presence has yet been found north of 55°N in Europe (*34*). This northern limit likely would represent the maximal extent of stable Neanderthal occupation. From ∼44 YBP onward, Neanderthals progressively retreated from their northeastern range (*35*), with the contraction accelerating after ∼38 YBP (*33*), ultimately leading to increasingly fragmented and isolated populations (*36*).

### The Neanderthal ancestry gradient direction was maintained during the Neolithic

As mentioned earlier, Neanderthal ancestry in NF is statistically lower than in HG but still distributed along a statistically significant Southeast-Northwest population gradient (Table 1). Because previous simulation studies have shown that genetic gradients can occur not only parallel to an expansion axis but also perpendicular to it, through genetic surfing (*37*), we assessed whether the empirical Neanderthal ancestry clines observed in HG and farmers could be obtained by multiple alternative demographic scenarios leaving similar signatures. We thus simulated four spatially explicit scenarios by adding an additional layer of farmer populations and alternatively varying the source of HG and farmer expansions, placing them either in the Southeast (Northeast Africa for HG and Anatolia for farmers) or the Southwest (Iberia for both HG and farmers). In these simulations, the expansion origins of both HG and farmers were arbitrarily defined, the goal being to approximate the general direction of their putative dispersal axis. We accounted for ongoing gene flow between African and Middle Eastern populations by incorporating the northern part of the African continent into our simulations. First, we investigated whether the observed Southeast-Northwest gradient of Neanderthal ancestry reflects the directional expansion of early HG. Our simulations revealed that, regardless of the assumed source (Southeast or Southwest), HG populations expanding into Europe consistently developed a spatial gradient of Neanderthal ancestry increasing with distance from their origin (Fig. 3). This pattern appeared in 90 to 96% of simulations (Table 2). In the two scenarios where the HG expansion originates in the Southwest (S2-WW and S4-WE), a Southeast-Northwest gradient emerges in only 3% of simulations. It means that the observed Southeast-Northwest gradient is compatible only with scenarios where HG expanded from the Southeast (scenarios S1-EE and S3-EW; Table 2), in line with archaeological evidence for modern human dispersal into Europe from Southwest Asia (*38*, *39*). Thus, our simulations demonstrate that the Paleolithic expansion of HG into Europe is a strong candidate for establishing the observed Southwest-Northeast gradient of Neanderthal ancestry. Note that while the observed gradient indicates the broad direction of the expansion (*15*, *40*), it cannot pinpoint its exact source, which may lie in unstudied or poorly sampled regions.

**Fig. 3.**
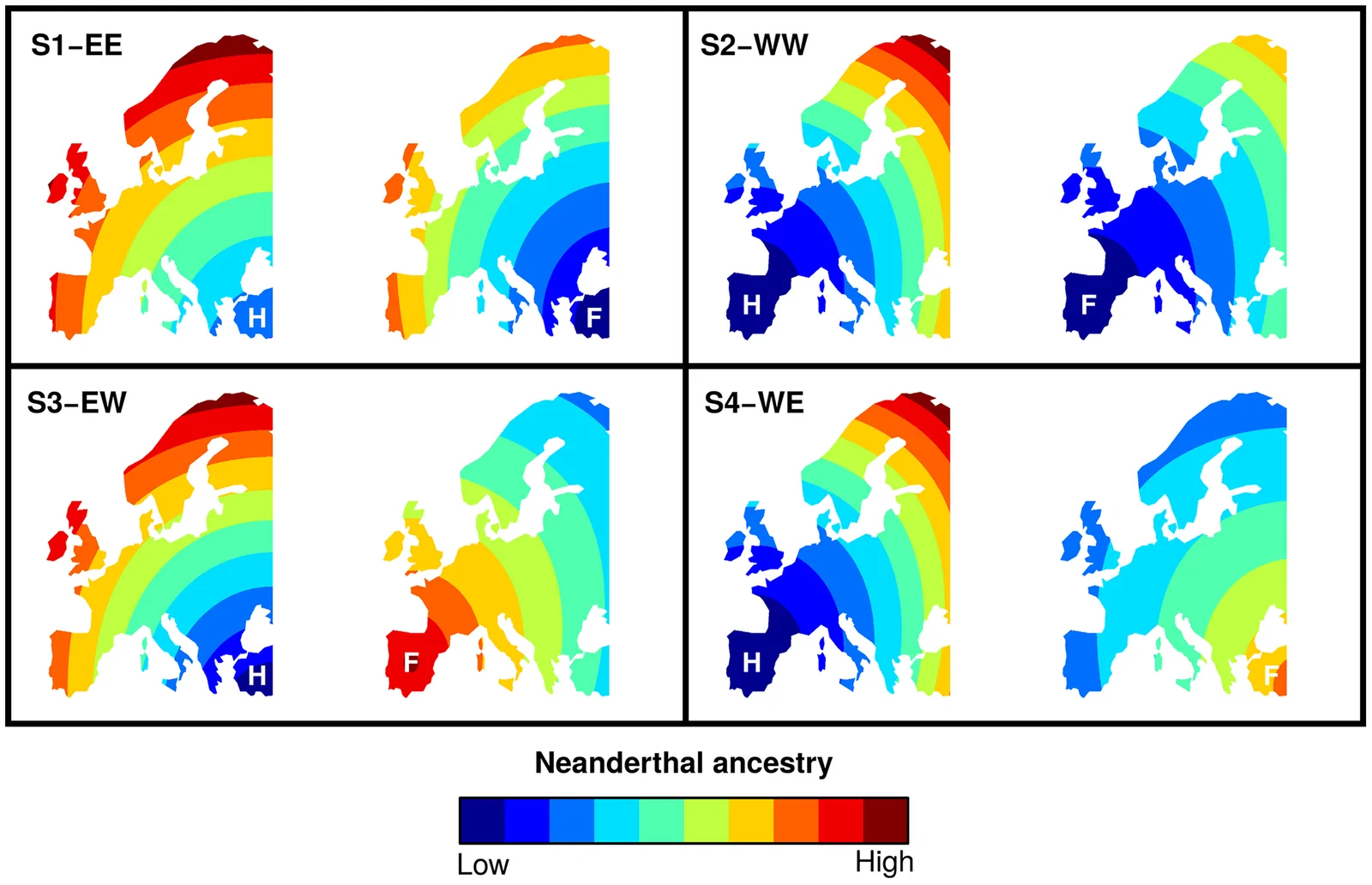
Extrapolated Neanderthal ancestry levels in HG and NF under four simulated expansion scenarios (S1 to S4). Extrapolation was based on a linear model for HG and a linear mixed model (LMM) for NF, fitted to the mean Neanderthal ancestry levels of each scenario. In each panel, the first map shows HG ancestry and the second map NF ancestry. White letters mark the reference point from which distances were measured (H, HG; F, NF). For NF, this always coincides with their expansion origin. For HG, it coincides with their own expansion origin in panels S2-WW and S4-WE, but with the NF origin in panels S1-EE and S3-EW, to allow distance to be calculated from a common reference point for both populations, as the source of HG in Northeast Africa is not represented in the map.

**Table 2.** Proportions of simulations producing a gradient of Neanderthal (NE) ancestry increasing with distance either from the respective expansion source or Anatolia (the presumed entry point of HG and NF into Europe). Results are shown separately for HG and NF under four expansion scenarios: S1(EE) both groups expand from their presumed origins (HG from Africa and NF from Anatolia); S2(WW) both expand from a hypothetical location in central Iberia; S3(EW) HG expand from Northeast Africa and NF from central Iberia; S4(WE) HG expand from central Iberia and NF from Anatolia. For each scenario, we also report the proportion of simulations in which HG exhibit higher mean Neanderthal ancestry than NF.

	Percentage of simulations			
Scenario	S1-EE	S2-WW	S3-EW	S4-WE
NE ancestry cline increasing in HG with distance from the HG source	0.90	0.96	0.90	0.96
NE ancestry cline increasing in NF with distance from the NF source	0.92	0.95	0.11	0.06
NE ancestry cline increasing in HG with distance from Anatolia	0.90	0.03	0.90	0.03
NE ancestry cline increasing in NF with distance from Anatolia	0.92	0.05	0.88	0.06
Higher NE ancestry in HG than in NF	0.57	0.96	0.07	0.37

Furthermore, our spatially explicit simulations show that the pattern of Neanderthal ancestry in farming populations, acquired through admixture with HG, depends not only on the origin and direction of the farmer expansion but also on those of the HG expansion. While the HG expansion defines the orientation of the gradient, the farmer expansion determines whether it increases or decreases. When farmers expanded along the same axis as HG (models S1-EE and S2-WW in Fig. 3), Neanderthal ancestry in farmers increased with distance from their expansion source, a pattern reproduced in 92 to 95% of simulations (Table 2). By contrast, when farmers expanded orthogonally to the HG expansion (Fig. 3 and figs. S3 and S4), the gradient reverses, with ancestry decreasing with distance from the farmer source (89 to 94% of simulations). For instance, when the farmer source is in the Iberian Peninsula and the HG source is in southeastern Europe, Neanderthal ancestry in farmers decreases with distance from Iberia and therefore increases with distance from Southeast Europe (Fig. 3 and fig. S3). Consequently, the observed increase of Neanderthal ancestry in farmers with distance from the Southeast is only compatible with the two scenarios in which HG themselves expanded from the Southeast (S1-EE and S3-EW; Table 2), regardless of where the farmer expansion began. This finding aligns with François *et al.* (*37*), who showed that successive expansions can maintain the spatial genetic pattern established during the initial colonization, provided that gene flow between populations is sufficient. In the case of Neanderthal ancestry, the similarity between pre- and post-Neolithic patterns reflects the farming populations “inheriting” an already established gradient. This highlights the importance of attributing observed gradients to the demographic events that originally generated them, rather than to subsequent population movements (*41*). Overall, our simulations indicate that the Neanderthal ancestry gradient was created by the initial HG expansion and maintained during the subsequent farmer dispersal through admixture.

### The temporal fate of Neanderthal ancestry in European populations

A central question regarding Neanderthal introgression in modern humans is its temporal fate. The proportion of Neanderthal ancestry in HG could have been as high as ∼6 to 10% in the first few generations after the admixture event and declined rapidly within the following 10 to 20 generations, due to purifying selection against weakly deleterious Neanderthal alleles (*10*, *11*, *42*). These alleles would have been efficiently purged from the HG gene pool because of their larger effective population size, leading to a rapid stabilization around present-day levels. However, postzygotic genomic (e.g., Dobzhansky-Müller) incompatibilities between Neanderthals and modern humans could have also contributed to the negative selection against Neanderthal-derived alleles (*43*, *44*). The paleogenomic dataset we analyzed nevertheless revealed a small but statistically significant decline of Neanderthal ancestry in HG (Table 1). In the lat55 scenario, which excludes the Neolithic expansion, simulations of neutral loci still replicated the slight temporal decrease in Neanderthal ancestry in HG without invoking any selective pressure (table S3). Our model begins after the initial purging phase, once Neanderthal ancestry has stabilized and simulates only its subsequent gradual change driven by neutral demographic processes. This suggests that genetic drift within structured HG populations can account for a slow gradual reduction of Neanderthal ancestry without invoking long-term selection. However, our simulations suggest that the markedly lower Neanderthal ancestry observed in post-Neolithic Europeans compared to pre-Neolithic groups is more consistent with the arrival of farmers and their admixture with HG, than with drift alone. This is supported by a 79% posterior probability for a scenario accounting for the Neolithic expansion compared to a scenario without it, based on an ad hoc ABC model choice.

All four simulated scenarios revealed an expected statistically significant difference in Neanderthal ancestry between HG and farmers (Welch’s *t* tests; table S4). Two scenarios show more Neanderthal ancestry in farmers than in HG, while two show the reverse. In S1-EE, the mean difference is 0.007 in favor of HG; in S2-WW, 0.026 in favor of HG; in S3-EW, 0.021 in favor of farmers; and in S4-WE, 0.009 in favor of farmers. Our simulations thus show that the starting location of the farmer expansion is critical to explain the decrease of Neanderthal ancestry in post-Neolithic populations because this expansion needs to start with a population having a lower-than-average amount of Neanderthal introgression. If the farmer expansion had begun in a region where Neanderthal ancestry was higher compared to other regions, then the mean difference in Neanderthal ancestry between HG and farmers would have shifted in favor of the farmers (e.g., scenarios S3-EW and S4-WE). Among the two scenarios that reproduce the observed Southeast-Northwest cline (S1-EE and S3-EW), only S1-EE, where HG and farmers expand in the same direction, resulted in higher Neanderthal ancestry in HG than in farmers, reproducing the observed pattern. This indicates that the Neolithic expansion drove the overall decline in Neanderthal ancestry levels while maintaining its gradient with the same orientation. This result is consistent with the origin of migrating early farmers in the Aegean area and western Anatolia (*19*), where pre-Neolithic populations carried less Neanderthal ancestry than their contemporaries in Central Europe (*45*, *46*). NF display an opposite temporal trend than HG, with a slow statistically significant increase in Neanderthal ancestry (Table 1), as also observed in the study of Quilodrán *et al.* (*15*). This increase can be explained by the increasing admixture between HG and farmers during the Neolithic transition (*23*). Because HG carried higher proportions of Neanderthal ancestry, their gradual admixture with farmers led to a slight increase of Neanderthal ancestry in farmers.

Overall, although our scenarios are extreme simplifications of the much more complicated genomic and migratory history of ancient humans (*25*), our simulations demonstrate that two consecutive population expansions along a Southeast-Northwest axis have the potential to reproduce both the observed clinal pattern of Neanderthal ancestry in European populations since the Paleolithic and the continent-wide reduction of Neanderthal ancestry during the Neolithic. Under the proposed model, this decline occurred through dilution, as initially proposed by Vernot and Akey (*47*) and may account for the lower levels of Neanderthal ancestry observed in present-day Europe compared to East Asia (*48*–*50*), assuming that a similar process did not take place in Eastern Eurasia.

### Three-population dynamic parameter estimation

On the basis of the scenario which produces more often the observed Neanderthal ancestry populational patterns (gradients and difference between HG and farmers; Table 2), we performed a parameter estimation using ABC. This scenario represents two successive expansion sources in the Southeast (scenario S1-EE) and the most probable northern limit of the Neanderthal range (55° latitude; table S2). The ABC analysis indicated that our three-population layered model was able to jointly reproduce the observed spatiotemporal Neanderthal ancestry patterns both in farmers and HG. It yielded a marginal posterior density *P* value of 0.69 for a tolerance rate δ = 0.01 (*P* value = 0.71 for δ = 0.005 and *P* value = 0.68 for δ = 0.03), thus allowing us to estimate key population dynamic parameters. Our model is therefore able to explicitly capture the population-specific characteristics of Neanderthal ancestry in two subsequent human expanding populations.

Figure 4 shows the prior and posterior distributions of each parameter with δ = 0.01, and table S5 shows the associated posterior distribution characteristics. The hybridization rate between Neanderthals and HG (γ_H_) is characterized by a well-defined (Fig. 4A) and narrow posterior distribution [95% highest density interval (HDI): 0.004 to 0.008], with a mode of ∼0.006. Cross-validation revealed a strong correlation between the pseudo-observed and inferred parameter values (*r* ≈ 0.83), together with a low relative bias (0.088) and root mean square error (RMSE = 0.001). In addition, the Factor2 value was 1, indicating that all parameter estimates fell within one-half to twice their true values. Together, these results demonstrate that the parameter can be inferred with high accuracy and robustness. This shows that the observed gradient of Neanderthal ancestry in HG developed under strong reproductive isolation between Neanderthals and early HG, with less than 0.8% of their contacts producing viable, fertile offspring, in accordance with estimates obtained from modern genomic data using a spatially explicit simulation approach modeling modern humans as a single population [0.5 to 0.8%, confidence interval (CI) between 0.33 and 1.3%] (*51*, *52*).

**Fig. 4.**
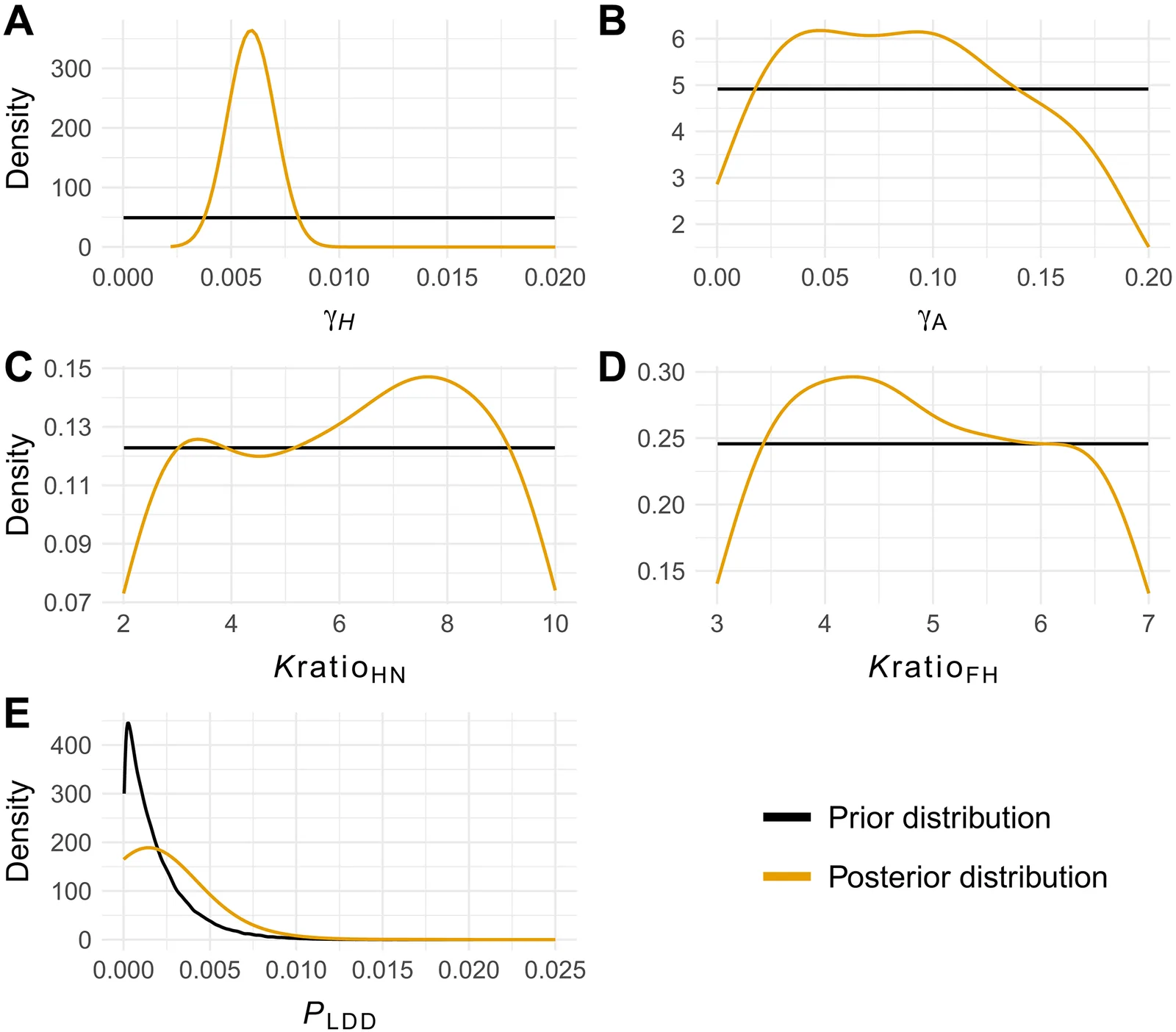
Prior and posterior distributions of the estimated parameters. The black line shows the prior distribution used in the simulations, and the yellow line the posterior distribution inferred from the ABC estimation. (**A**) Hybridization rate between Neanderthals and HG (γ_H_). (**B**) Assimilation rate of HG into NF (γ_A_). (**C**) Ratio of HG to Neanderthal effective population size (**K*ratio*_*HN*_). (**D**) Ratio of farmer to HG effective population size (**K*ratio*_*FH*_). (**E**) Proportion of farmer migrations occurring as long-distance dispersals (*P*_LDD_).

The estimated assimilation rate of HG into farmers (γ_A_), required to maintain the observed Neanderthal ancestry gradient in European populations, shows a significant but moderate correlation between pseudo-observed and inferred values (*r* ≈ 0.23; table S5). This estimate should therefore be interpreted with caution given its considerable uncertainty, reflected by a broad 95% HDI (0.002 to 0.181; table S5), large relative bias (1.93), and RMSE (0.061). Nevertheless, the point estimate (0.049) is very close to the ∼0.046 reported by Tsoupas *et al.* (*23*) based on molecular diversity computed on 67 high quality paleogenomes. It gives an order of ∼5% of contacts between HG and farmers resulting in gene flow from HG to farmers. This limited gene flow may reflect ecological, economic, or cultural segregation (*53*). Notably, the HG-farmer assimilation rate is estimated to be ∼15 times higher than the Neanderthal-HG hybridization rate (table S5), in accordance with the strong interspecific reproductive barriers between Neanderthals and HG, which obviously do not exist between HG and farmers.

The remaining parameters could not be estimated accurately, as indicated by the lack of significant correlations between true and inferred values in the cross-validation analysis (table S5). Nevertheless, allowing them to vary was essential to account for their uncertainty when estimating admixture rates. Their posterior distributions should therefore be interpreted as broad approximations rather than precise estimates. The ratio of the effective population sizes of HG and Neanderthals (**K*ratio*_*HN*_) had a 95% HDI of 2.3 to 9.7 (Fig. 4C), with high relative bias (0.48) and RMSE (2.52). The ratio of the effective population sizes of farmers and HG (**K*ratio*_*FH*_) had a 95% HDI of 3.14 to 6.82 (Fig. 4D), with lower relative bias (0.22) but relatively high RMSE (1.4). The proportion of long-distance dispersal (LDD) during the Neolithic expansion was concentrated near zero (95% HDI: 0 to 0.008), with high relative bias (1.81), an RMSE of 0.002, and a low Factor2 (0.481). In addition, a set of supplementary simulations, using the same simulation framework and parameter priors but without simulating LDD, was able to replicate the observed Neanderthal ancestry gradient in farmers, as the marginal density *P* value of the simulations was 0.68, which means that the observed gradient can be formed even in the absence of LDD. These results indicate that LDD had minimal impact on the gradient and overall levels of Neanderthal ancestry in farming populations, although it has been shown to reduce introgression (*54*). LDD may have played a more substantial role in increasing the speed of the farmer expansion, as suggested by Tsoupas *et al.* (*23*).

### Model strengths, limitations, and perspectives

The strength of our approach lies in its spatially explicit, three-layered design applied to an extensive paleogenomic dataset. This framework enabled us to define spatiotemporal patterns of Neanderthal ancestry and use them for performing demographic inference. By incorporating introgression from a third population, our framework extends the previous version of SPLATCHE3 (*26*), which was restricted to two interacting lineages. This allowed us to model Neanderthal ancestry across space and time through two successive expanding populations with admixture, pre-Neolithic HG, and NF and to compare alternative scenarios against a large paleogenomic dataset to identify the processes underlying the observed cline of Neanderthal ancestry in Europe. This new version of SPLATCHE3 is the first spatially explicit demographic simulator to model three populations interacting across a realistic two-dimensional landscape. This greatly expands its applications, enabling the investigation of more complex demographic histories, such as successive Paleolithic, Neolithic, and Bronze Age expansions in Europe or the peopling of the Americas. Although the current implementation allows only two of the three populations to coexist at any given time, future developments could enable all three populations to interact simultaneously.

While powerful, our framework is computationally demanding and relies on simplifying assumptions, consistent with our aim of investigating broad chronological demographic scenarios encompassing two major human migration events rather than fine-scale demographic processes. Nevertheless, only the Neanderthal-HG hybridization rate was estimated with high precision, whereas the remaining parameters showed broad posterior distributions and low inference accuracy. This limited accuracy likely reflects both the simplified nature of our demographic models, which cannot capture all aspects of human population history and the limited informativeness of the summary statistics used for ABC. The low *R*^2^ values of the regression models indicate that a substantial proportion of the variance remained unexplained, suggesting that some features of the ancestry gradients were not fully captured by our model and may result from smaller-scale or regional processes (*55*). For instance, we assumed constant demographic and migratory parameters for Neanderthals, HG, and farmers, whereas geographic features can facilitate or impede migration and strongly influence admixture patterns. Future work should thus examine the effects of regional heterogeneity (*56*, *57*) and fluctuating population sizes (*58*–*60*). For example, faster dispersal along major waterways (*61*) may accelerate colonization, whereas mountain ranges can act as barriers shaping both genetic diversity and admixture patterns (*62*). Incorporating such geographic features would, however, require calibrating additional demographic parameters, including migration rates and dispersal distances, thereby increasing model complexity and computational demands. Adding poorly constrained parameters could introduce noise and reduce the interpretability of the results.

Although our simulations suggest a minimal Neanderthal presence north of 55° latitude, this pattern could potentially be refined by modeling variable population densities, e.g., larger in the south, lower in the north, to investigate whether it fits better the observed ancestry patterns. Because our framework now considers neutral loci, it likely underestimates the frequency of admixture before the purging of Neanderthal DNA introgressed in human genomes during the first 10 to 20 generations after hybridization (*10*, *11*). Incorporating this effect would need to include selection parameters in the model, which would add another level of complexity. Likewise, the model cannot address whether reproductive isolation between Neanderthals and HG was pre- or postzygotic. For the sake of simplicity, we also assumed a constant HG-farmer assimilation rate, despite evidence that it increased during the Neolithic transition (*23*, *63*) and did not account for asynchronous or multiwave expansions nor for different demographic and migratory history between the Danube and Mediterranean route of the Neolithic expansion (*20*). Also, our model does not include minor or dead-end modern human migrations that left little genetic trace in later populations (*64*).

Last, an important event not included in our model, but warranting further investigation, is the Last Glacial Maximum (LGM; 19 to 27 YBP), a period during which the fate of human populations remains poorly understood. For example, it is unclear to what extent HG retreated to southern refugia and whether their distribution was continuous [e.g., (*58*)] or fragmented [e.g., (*65*, *66*)]. This period is particularly relevant because it falls between the two events examined here: the initial settlement of Europe by HG and the Neolithic transition. Understanding how Neanderthal ancestry gradients persisted or were reshaped after the LGM would provide critical insights.

### Conclusion

Our original simulation framework represents a step forward toward developing more detailed scenarios for investigating how demographic variation, migration, and population interactions have shaped spatiotemporal patterns of genomic ancestry. As paleogenomic data continues to accumulate with improved sequencing quality, this iterative approach has the potential to substantially refine our understanding of ancient population dynamics and enable more precise demographic inferences about major events that marked the history of our ancestors across different periods. Our study highlights the key role of demographic processes during range expansions in shaping patterns of archaic introgression, providing alternative explanations to selection or uneven hybridization, although these mechanisms are not mutually exclusive. More broadly, our study demonstrates the potential of spatiotemporal ancestry patterns to reconstruct expansion directions and to estimate demographic parameters such as the hybridization rate. Although the shared direction of the two expansions had previously been inferred from archaeological evidence, we independently recovered the same pattern from paleogenomic data alone, illustrating the potential of genomic data to reconstruct past demographic history. Notably, our results show that interspecific introgression can also illuminate intraspecific demographic processes. The framework can be readily extended to later periods of Prehistory, such as the Bronze Age steppe migrations (*18*, *67*), or modified to study introgression from other archaic groups like Denisovans, as well as to trace the genomic composition of human populations through time. Beyond the case studied here, this approach offers broad applicability for investigating species interactions across diverse research contexts.

## MATERIALS AND METHODS

### Three-population layered spatially explicit simulations

We used SPLATCHE3 (*26*) to simulate interactions between Neanderthals and HG and the resulting patterns of Neanderthal ancestry. The program models two overlapping population layers on a spatially explicit ASCII map of Europe divided into cells, each containing two demes with distinct demographic parameters. Layers interact through user-defined settings. Simulations proceed forward in discrete generations for demography, followed by a backward coalescent step that generates molecular diversity in samples from various locations and times, as defined by the user. More specifically, we used a modified version of SPLATCHE3 [published in (*24*) and available on Zenodo; https://zenodo.org/records/21829168] that includes three new features. First, we extended the original two-population framework to three populations (A, B, and C), with the condition that only two coexist at any given time. Once one of the initial populations (A) goes extinct through competition with the other (B), a third population (C) can be introduced into the empty layer and interact with the surviving one (B). In this study, these correspond to Neanderthals, pre-Neolithic HG, and NF: Neanderthals and HG were the initial populations, while farmers appeared later, derived from HG in Anatolia after Neanderthal extinction. SPLATCHE3 then outputs the proportion of loci in HG and farmer samples that originated from Neanderthals (i.e., ancestry proportion). These proportions can be estimated with F4-ratios (*68*) computed on real paleogenomic data, with which it has been shown to be highly correlated (Pearson’s *r* > 0.75) (*69*). The F4-ratio is the statistic used to estimate Neanderthal genomic contribution to modern humans in the observed dataset. It is a robust estimator for heterogeneous coverage data, although lower sequencing coverage tends to increase its uncertainty. Second, we allowed admixture models to change at a predefined generation, switching from hybridization (Neanderthal-HG) to assimilation (HG-farmers). In the hybridization model, contacts generate hybrid individuals assigned to one of the two parental species, resulting in bidirectional gene flow (*70*, *71*). In the assimilation model, genes from one population are incorporated into the other population, resulting in unidirectional gene flow, here from HG to farmers. Gene flow frequency is controlled by a parameter γ: In the hybridization model, γ_H_ represents the proportion of individuals producing hybrid offspring in the next generation; in the assimilation model, γ_A_ represents the proportion of individuals from one population incorporated into the other (see the SPLATCHE3 manual, www.splatche.com). Third, competition between layers could switch from density-dependent (*16*, *41*) to a user-specified rate (α). The intensity of competition determines the duration of cohabitation. Because the duration of Neanderthal-HG coexistence in Europe is uncertain (*6*, *7*), we modeled their competition as density dependent, which simplified simulations without affecting HG sampling. For HG-farmer interactions, however, density-dependent competition caused HG to disappear too early, preventing dataset recovery. Cohabitation time being primarily determined by population sizes and α, we set α to a fixed value during the early farmer expansion to allow prolonged coexistence and then switched back to density-dependent competition 10 generations after the date of the last HG sample, ensuring HG extinction during the Neolithic. To maximize simulation success, α was estimated individually for each simulation relative to the ratio of farmer to HG carrying capacity (**K*ratio*_*FH*_). The procedure was as follows: (i) 200,000 initial simulations were run, and only those reproducing the observed dataset (correct samples in time and space) were retained; this corresponds to the “demographic filter” in the study of Rio *et al.* (*68*); (ii) **K*ratio*_*FH*_ and α from the retained simulations were used to fit a linear model (α ∼ **K*ratio*_*FH*_) with the lm() function in R; (iii) α for each subsequent simulation was predicted from its **K*ratio*_*FH*_ using predict.lm(). This yielded α values between 0.113 and 0.267, very close to the one estimated in Tsoupas *et al.* (*23*) which used a paleogenomic dataset specifically designed to study the Neolithic transition along the Danube route (α = 0.15 to 0.327).

The simulation framework followed the study of Quilodrán *et al.* (*16*), using a map of Europe, the Middle East, and the Northern part of Africa divided into 75 × 53 cells of 100 × 100 km (fig. S3). The northern part of the African continent was included in our simulations to account for gene flow between African populations lacking Neanderthal ancestry and populations in the Middle East. The initial expansion sources were set at: (i) an arbitrary point in Northeast Africa (30° and 34°) for the HG layer, representing the out-of-Africa expansion following Quilodrán *et al.* (*15*); and (ii) an arbitrary point in Anatolia (37° and 36°) for the farmer layer, consistent with paleogenomic evidence for the European Neolithic expansion starting from this area (*19*, *65*). Sample coordinates were converted to WGS84 Plate Carree format using the proj4 R package (*72*), as required by SPLATCHE3 to map samples onto the ASCII simulation grid. The lower-left map corner was set at 3,201,000, −1,647,000 (WGS84 Plate Carree). Simulations started at 70,000 YBP to allow Neanderthals sufficient time to populate Europe and reach demographic equilibrium before modern humans appeared. With a generation time of 25 years (*73*), this corresponded to 2800 simulated generations. Because our focus was on modern human expansions, earlier generations were omitted to improve computational efficiency. The HG expansion into Europe was set at generation 800 [∼50,000 YBP (*74*)] and the farmer expansion at generation 2380 [∼10,500 YBP (*21*)].

The mean ages of observed genomes were converted to generations since the start of the simulation. Constant parameters followed the study of Quilodrán *et al.* (*16*), and Table 3 lists them, as well as those that varied across simulations and their prior ranges. All used parameters can be found in the setting files provided for each simulated scenario in Zenodo (https://zenodo.org/records/21829168). For each genome, we simulated 50 independent, neutrally evolving genomic loci to estimate Neanderthal ancestry. This number balances computational efficiency with accuracy (*68*), as shown in fig. S4.

**Table 3.** Prior value ranges of parameters that were varying between simulations and values of the fixed parameters.

Parameter	Ranges of the prior distributions/fixed value
Neanderthal growth rate (*r*_NE_)	0.8
Neanderthal migration rate (*m*_NE_)	0.3
Neanderthal effective population size (*K*_NE_)	200
Hybridization rate between Neanderthal and HG (γ_H_)	0.0–0.02
HG growth rate (*r*_HG_)	0.8
HG migration rate (*m*_HG_)	0.2
HG effective population size (*K*_HG_)	400–2,000
Assimilation rate of HG into NF (γ_A_)	0.0–0.2
NF growth rate (*r*_NF_)	0.8
NF migration rate (*m*_NF_)	0.3
NF effective population size (*K*_NF_)	1,200–14,000
Proportion of long-distance dispersals (*P*_LDD_) in farmers	0–0.025
HG to Neanderthal effective population size ratio (**K*ratio*_*HN*_), defined as *K*_HG_*/K*_NE_	2–10
NF to HG effective population size ratio (**K*ratio*_*FH*_), defined as *K*_NF_*/K*_HG_	3–7

Interbreeding between Neanderthals and HG occurred in any cell of co-occurrence, with a probability defined by the hybridization rate (γ_H_). Assimilation of HG into farmer demes was governed by the assimilation rate (γ_A_), which applied where both populations coexisted. These rates reflect the probability that contact between two populations results in gene flow. For γ_H_, the prior range was 0 to 0.02, and for γ_A_, the prior range was 0 to 0.2 (*51*, *63*). Assimilation was unidirectional (HG into farmers), and the switch from hybridization to assimilation was set one generation before the emergence of farmers (generation 2379).

The ratio of HG to Neanderthal effective population size (**K*ratio*_*HN*_) ranged between 2 and 10 (*69*), while the ratio of farmers to HG size (**K*ratio*_*FH*_) ranged between 3 and 7 (*23*). For each simulation, both ratios were drawn from uniform distributions. Neanderthal effective size was fixed at 200 individuals, from which HG and farmer sizes were derived: HG size = 200 × **K*ratio*_*HN*_; farmer size = HG size × **K*ratio*_*FH*_. As SPLATCHE3 requires haploid counts, all effective sizes were doubled to obtain the number of gene copies used in the simulations.

Neanderthals and HG migrated in a two-dimensional stepping-stone manner (*75*). Farmers also migrated primarily in a stepping-stone fashion but with a small probability (*P*_LDD_) of LDD events, ranging from 0 to 0.025. *P*_LDD_ values were drawn from an exponential distribution to explore more often lower values, as Tsoupas *et al.* (*23*) showed that only a small percentage of the total migrations took place over long distances. In SPLATCHE3, migration distances for LDD can only be drawn from a gamma distribution. For the present analysis, the distances were sampled from a gamma distribution with shape β = 1.209 and rate λ = 0.15046, following Rio *et al.* (*68*).

### Paleogenomic dataset

Paleogenomic data were retrieved from the AADR database v50.0 (*28*), which contained 10,391 genomes. We retained only European genomes (longitude < 34°) belonging to Paleolithic/Mesolithic HG or Neolithic/Chalcolithic farmers (NF). The assignment of individuals to these two groups was based primarily on the AADR “GroupID” column or on the mean age of the genome (all genomes older than 15,000 years were classified as HG). In cases of uncertainty, we relied on the information provided in the original publication describing the sequencing of the ancient individual. For genomes represented by multiple versions, we kept the one with the highest SNP coverage. We filtered out putatively contaminated genomes by using the contamination warning estimated through linkage disequilibrium (*76*): Entries flagged as “Model_Misspecified” or “Very_High_Contamination,” or with “contam/possible.contam” in their GroupID, were excluded. We also excluded genomes lacking geographic coordinates. For dating, we used the mean age (YBP) provided in the database, corresponding either to the midpoint of the 95.4% CI calibrated radiocarbon date or the midpoint of the archaeological context range.

#### Estimation of Neanderthal ancestry

Neanderthal ancestry was estimated using F4-ratios (α_f4_) as described by Reich *et al.* (*77*). The F4-ratio, defined as α_f4_ = F4(A, O, X, C)/F4(A, O, B, C), measures the proportion of ancestry in a test genome (X) derived from population B, relative to a sister population C. Following Petr *et al.* (*45*), we used Dinka from eastern Africa as the unadmixed sister population (C), the Altai Neanderthal as sister population (A) of Vindija Neanderthal (B), and chimpanzee as outgroup (O). These groups are available in the AADR database as “Altai_Neanderthal.DG,” “Vindija_Neanderthal.DG,” “Dinka.DG,” and “Chimp.REF.” F4-ratios were computed with ADMIXTOOLS 2 (*78*), and genomes with nonsignificant values (|*Z*| < 3) were excluded. We refer to the α_f4_ as Neanderthal ancestry. After filtering, 715 genomes were retained (102 HG and 613 farmers), ranging in age from ∼35,000 to ∼4000 YBP. Genome metadata are provided in data S1, and sampling locations are shown in Fig. 2.

#### Summary statistics used for the ABC analysis

The observed dataset was aggregated to match the simulated samples. By converting (i) each individual’s age into the corresponding simulated generation and (ii) its sampling coordinates into the WGS84 Plate Carrée projection, we identified individuals that would be sampled from the same deme in the same simulated generation. Genomes from the same population group (HG or farmers), which would be sampled in the same simulated deme and generation (25-year intervals), were grouped together, and their Neanderthal ancestry was averaged, yielding 514 population samples (90 HG and 424 farmers; data S2). We then estimated the mean Neanderthal ancestry in the whole dataset and separately in HG and in farmers, with these values being used as summary statistics in the ABC (*27*) analysis (see below).

For each sample, geographic distance from the source of the corresponding expansion (fig. S3) was calculated with the gmt R package (https://rdrr.io/cran/gmt/). In NF, distance from the expansion source (*d*_NF_) was correlated with sample age in generations (Pearson’s *r* = 0.57, *P* value < 2 × 10^−16^), with more distant samples tending to be younger. To account for this, farmer samples were divided into three periods of ∼84 generations each, and Neanderthal ancestry was modeled as a function of *d*_NF_ and age using a linear mixed model (LMM; Table 1). The period was included as a random slope and intercept, with linear spatial autocorrelation. LMMs were fitted with the nlme R package (*79*). In HG, distance from the expansion source (*d*_HG_) was not correlated with age (Pearson’s *r* = 0.06, *P* value = 0.6), so Neanderthal ancestry was modeled with a simple linear model including *d*_HG_ and age (Table 1). In both models, Neanderthal ancestry was log-transformed to ensure normally distributed residuals. The coefficients from these models (Table 1), along with mean Neanderthal ancestry in HG and farmers, were then used as statistics in the ABC analysis.

### Impact of the northern limit of Neanderthal habitat on the Neanderthal ancestry gradient

We tested whether the northernmost limit of Neanderthal habitat in Europe influenced the observed Neanderthal ancestry gradient in HG. This analysis relied on ABC model choice to compare scenarios and assess their ability to reproduce the observed gradient. We simulated four scenarios with different northern habitat limits (fig. S1): 50° (“lat50”), 55° (“lat55”), 60° (“lat60”), and 65° (“lat65”). The 55° latitude reflects the generally accepted northern limit of Neanderthal occupation (*30*, *34*). Because adaptability to colder climates was not a limiting factor for their survival further north (*31*, *80*) and preservation issues cannot be ruled out (*81*), we explored the region south and north of 55° by dividing it into equal latitudinal bands of 5° each. Only Neanderthals and HG were simulated, with 400,000 runs per scenario. For each simulation, Neanderthal ancestry proportions in HG were fitted with the linear model described in Table 1A, and the resulting intercept, distance, and age coefficients, together with mean Neanderthal ancestry in HG, were used as summary statistics.

Goodness of fit between simulated and observed data was assessed with ABCtoolbox2 (*82*) by computing marginal density *P* values under the null hypothesis that simulations matched the observed statistics. Euclidean distances were calculated, and a tolerance δ of 0.005, 0.01, or 0.03 (corresponding to 2000, 4000, or 12,000 retained simulations) was applied to test robustness to tolerance level.

In addition, for each scenario, we estimated the proportion of simulations which produced a gradient of NE ancestry increasing in HG with distance from the expansion source, the proportion of simulations which produced a gradient of NE ancestry in HG decreasing with time, and the proportion of simulations that produced both such gradients. This was done to assess whether the simulations could reproduce the overall observed trend and to determine which scenario most frequently generated a gradient similar to the observed one.

Model choice was performed with the abcrf R package (*83*), which implements a random forest approach with strong discriminative power and relatively low simulation requirements. The abcrf R package estimates the posterior probability of the scenario chosen by the most trees. We tested different forest sizes and retained 2000 trees, as larger forests did not further reduce misclassification error.

### Impact of HG and farmer expansion directions on the Neanderthal ancestry gradient

Because genetic clines may differ from the axis of population expansion (*37*, *84*), we examined the effect of the direction of the two consecutive modern human expansions on the observed gradient of Neanderthal ancestry in Europe: the arrival of HG and the later migration of farmers. Using simulations, we compared the gradients produced when both groups expanded from the Southeast (the presumed entry point of both HG and later farmers into Europe) toward the Northwest versus the gradients when one or both groups expanded from a hypothetical entry point in the Iberian Peninsula toward the Northeast. We performed 10,000 simulations for each of four scenarios differing in the expansion origins of HG and farmers:

1) S1-EE: Both HG and farmers expand from the Southeast, HG from a chosen location in Northeast Africa and farmers in Anatolia (fig. S5A).

2) S2-WW: Both HG and farmers expand from the Southwest, in the Iberian Peninsula (fig. S5B).

3) S3-EW: HG expand from the Southeast (Northeast Africa) and farmers from the Southwest (Iberian Peninsula; fig. S5C).

4) S4-WE: HG expand from the Southwest (Iberian Peninsula) and farmers from the Southeast (Anatolia; fig. S5D).

For each scenario, we used simulated Neanderthal ancestry proportions to fit the same linear (HG) and LMMs (farmers) applied to the observed data (Table 1). We then estimated, separately for HG and farmers, the proportion of simulations of each scenario in which Neanderthal ancestry increased with distance from the respective starting location of each expansion or with distance from Anatolia (the presumed entry point of HG and farmers into Europe) (Table 2). Last, we estimated for each scenario the proportion of simulations in which HG had higher NE ancestry than farmers (Table 2) and the mean simulated difference in Neanderthal ancestry between HG and farmers (table S4).

### Population dynamic parameters shaping the Neanderthal ancestry gradient

We assessed whether levels of gene flow between Neanderthals and HG and between HG and farmers and the relative effective sizes of the three groups contributed to the observed Neanderthal ancestry gradient, as these factors are known to influence introgression patterns (*16*). We inferred the parameter values most compatible with the observed levels and gradient of Neanderthal ancestry by using 200,000 simulations of HG and farmer expansions from arbitrarily chosen locations representing their presumed origins (Northeast Africa for HG and Anatolia for farmers; fig. S3) with the three-population version of SPLATCHE3.

Parameter estimation was carried out with ABCtoolbox2, which computes the minimized Euclidean distance between simulated and observed summary statistics. The closest simulations are retained, and an ABC-GLM regression adjustment (*85*) is applied to their parameter values to estimate posterior distributions. As summary statistics, we used mean Neanderthal ancestry in HG and farmers and the coefficients of the respective linear models (Table 1, bold), for a total of eight statistics.

The model fit was assessed as in the analysis of the latitude of Neanderthal habitat. To test robustness to the tolerance rate (δ), we retained 1000, 2000, and 6000 simulations (δ = 0.005, 0.01, and 0.03, respectively). The posterior distribution of each parameter was compared with the respective prior, and posterior modes, means, and 95% HDIs were estimated. Here, the posterior modes are used as point estimates. For the two population ratios (**K*ratio*_*HN*_ and **K*ratio*_*FH*_), we also estimated a two-dimensional posterior distribution and used Markov chain Monte Carlo sampling to estimate the probability that **K*ratio*_*HN*_ is larger than **K*ratio*_*FH*_.

To evaluate the precision of the parameter inference, we used a cross-validation standard approach (see below), by computing the relative bias, the RMSE, and the Factor2 of the posterior mode. In addition, we computed the correlation between the inferred point estimate given by the posterior’s mode and the pseudo-observed parameter value.

For each simulation retained by the ABC, the simulated summary statistics were treated in turn as pseudo-observed values with known parameters, and posterior estimates were obtained from the remaining simulations through an ABC estimation. Relative bias was then calculated as: $\frac{1}{n}\sum_{i=1}^{n}\frac{|\mu_{i}-x_{i}|}{x_{i}}$ and the RMSE as $\sqrt{\frac{\sum_{i=1}^{n}{\left(\chi_{i}-\mu_{i}\right)}^{2}}{n}}$, where *n* is the number of pseudo-observations, *x*_*i*_ the pseudo-observed parameter value, and μ_*i*_ the posterior mode for that replicate. The Factor2 is defined as the proportion of estimated mode values μ_*i*_ being between 50 and 200% of the pseudo-observed parameter value *x*_*i*_. We also computed the average Pearson correlation coefficient (*r*) between all μ_*i*_ and *x*_*i*_.

To assess the importance of LDD in shaping the gradient of Neanderthal ancestry in farmers, we performed an additional set of 100,000 simulations, using the same simulation framework and parameter priors but without using LDD. The fit of these simulations to the observed data was assessed in the same way described earlier for the analysis of the latitude of Neanderthal habitat.

We also performed an additional set of 100,000 simulations, using the same simulation framework and parameter priors as for the parameter estimation, simulating a scenario where the Neolithic transition was purely cultural. This means that no farmer expansion was simulated and the farmer samples were drawn directly from the HG layer. We used an ABC analysis to compare this scenario to the scenario that included the Neolithic expansion, to investigate if the observed difference in NE ancestry between the pre- and post-Neolithic European populations could be the result of drift alone or if the arrival of farmer populations resulted in the dilution of NE ancestry.

Last, to demonstrate that simulating a larger number of loci does not substantially affect the estimated levels of Neanderthal ancestry, we performed an additional 20,000 pairs of simulations. Within each pair, both simulations used identical parameter values, but one simulated 50 independent, neutrally evolving loci, whereas the other simulated 500 loci. For each simulation, we estimated the mean Neanderthal ancestry and compared the resulting paired values (fig. S4).
