# Supplementary material for: Tracing Neanderthal ancestry patterns through successive population expansions in Europe
Source: Sci Adv. 2026 Nov 11;12(46):eaeg6161. doi: 10.1126/sciadv.aeg6161 (PMC13754034; doi:10.1126/sciadv.aeg6161)
Supplement: Supplementary file 1 [file sciadv.aeg6161_sm.pdf]

Science Advances
AAAS

Supplementary Materials for

Tracing Neanderthal ancestry patterns through successive population expansions in Europe

Alexandros Tsoupas *et al.*

Corresponding author: Mathias Currat, mathias.currat@unige.ch

Sci. Adv. **12**, eaeg6161 (2026)
DOI: 10.1126/sciadv.aeg6161

The PDF file includes:

Figs. S1 to S5
Tables S1 to S5
Legends for data S1 and S2

Other Supplementary Material for this manuscript includes the following:

Data S1 and S2

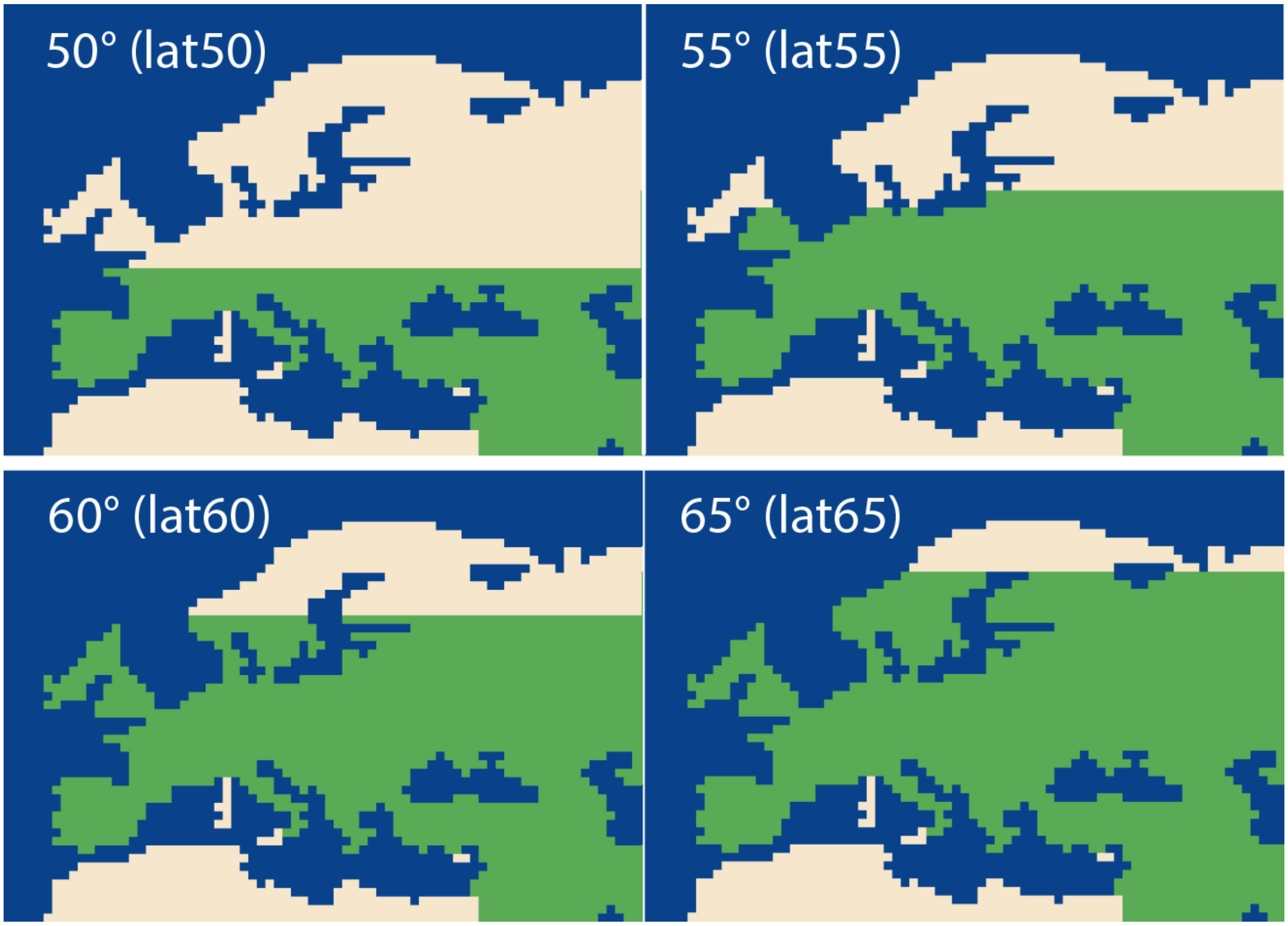

Supplementary Figure S1. The four tested northern limits of Neanderthal habitat. Green areas indicate regions habitable by Neanderthals, white those considered uninhabitable. Scenario names are indicated in parentheses in each panel. The four maps were generated using the SPLATCHE3 program, freely available at www.splatche.com.

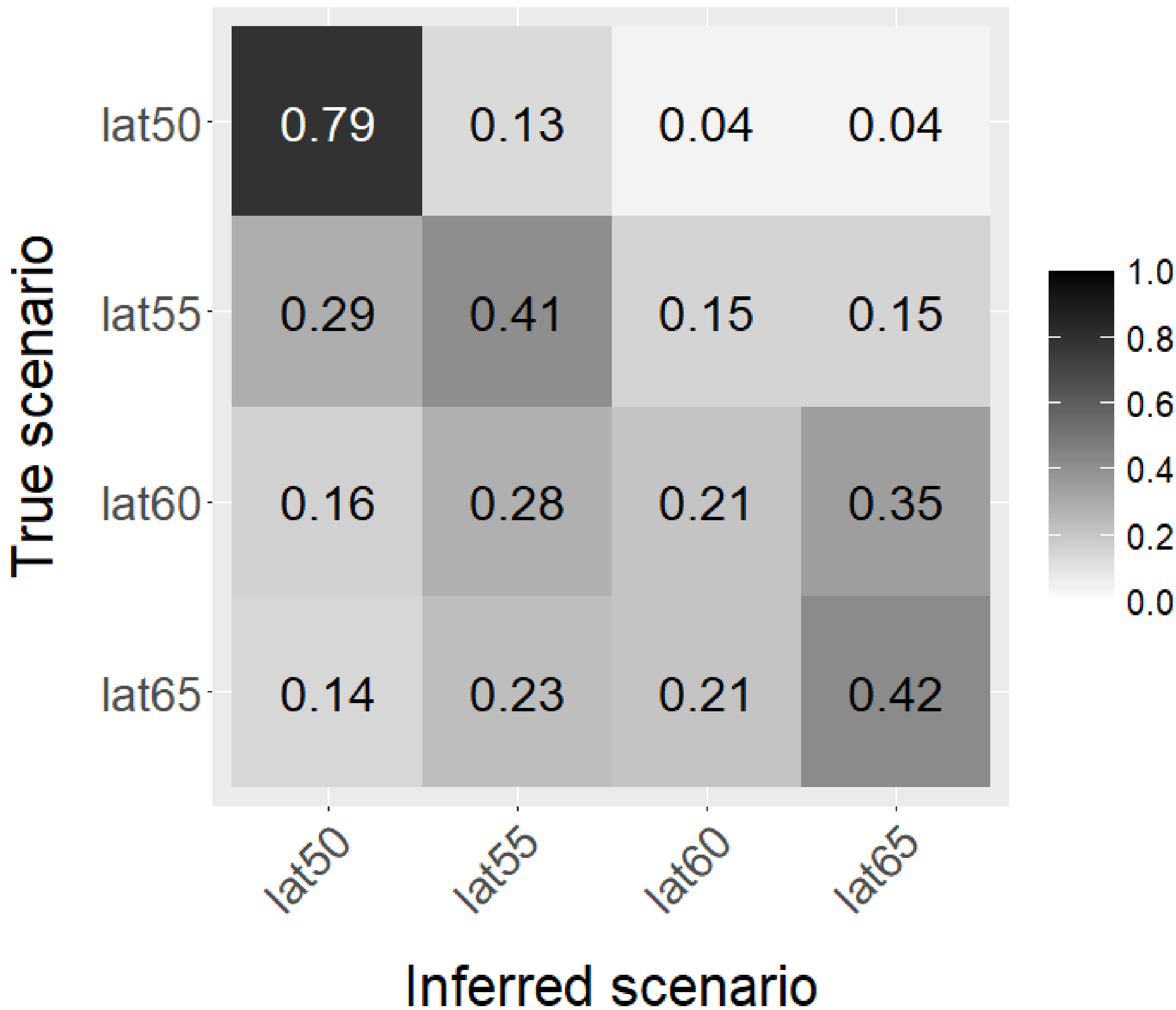

Supplementary Figure S2. Confusion matrix of the four investigated scenarios of Neanderthal habitat northern latitudinal limits. Calculation performed with the abcrf R package (ABC-random forest approach; *83*), using 2,000 trees and 400,000 simulations per scenario. Each row represents simulations from a given scenario (the 'true' scenario), while the columns indicate the proportion of those simulations that ABC attributed to each of the studied scenarios (the 'inferred' scenarios).

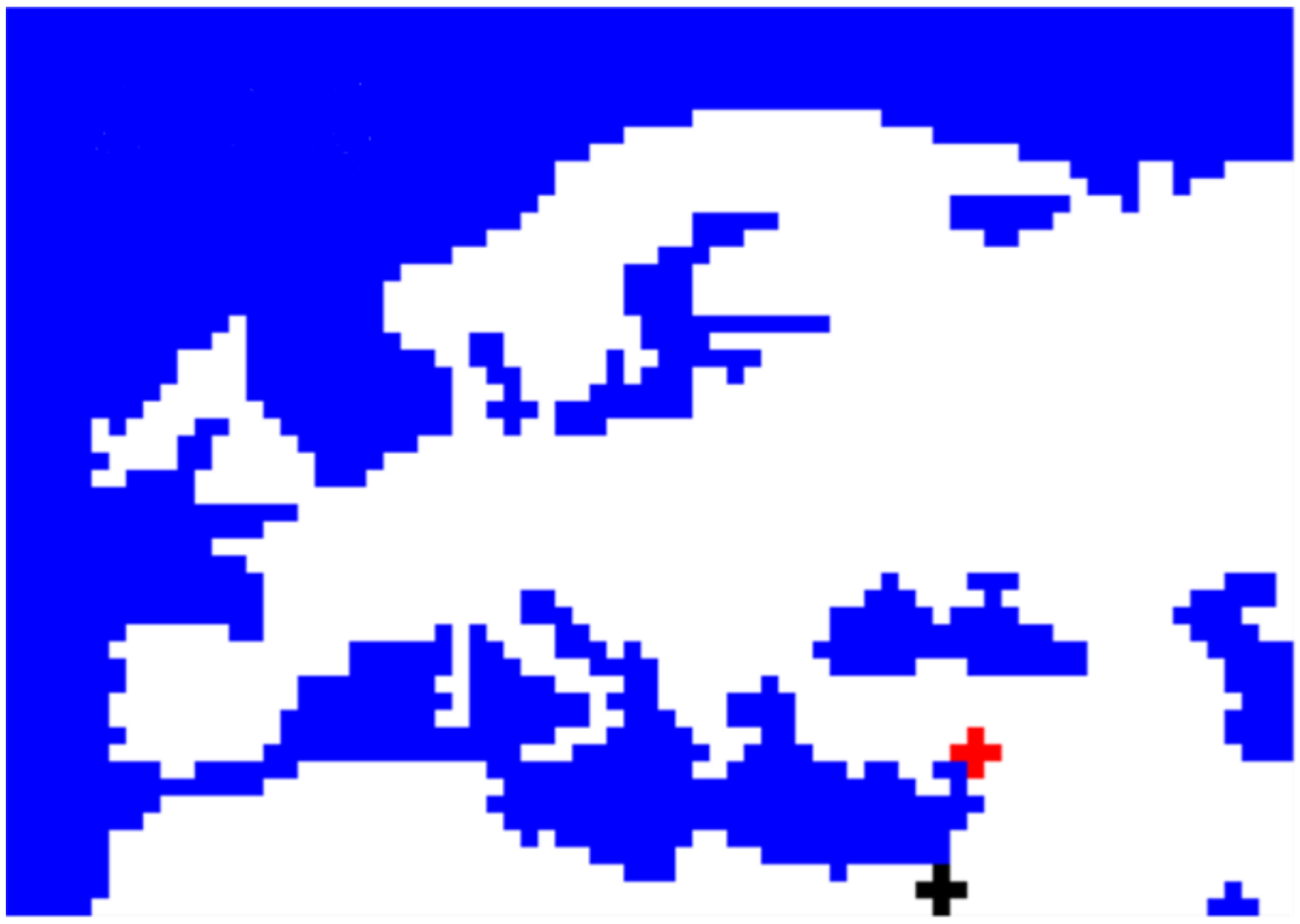

Supplementary Figure S3. **The locations chosen as starting points for the successive modern human expansions in the simulations for parameter estimation.** Black = pre-Neolithic Hunter-gatherer (HG) deme used as source, Red = Neolithic Farmer deme used as source. The map was generated using the SPLATCHE3 program, freely available at www.splatche.com.

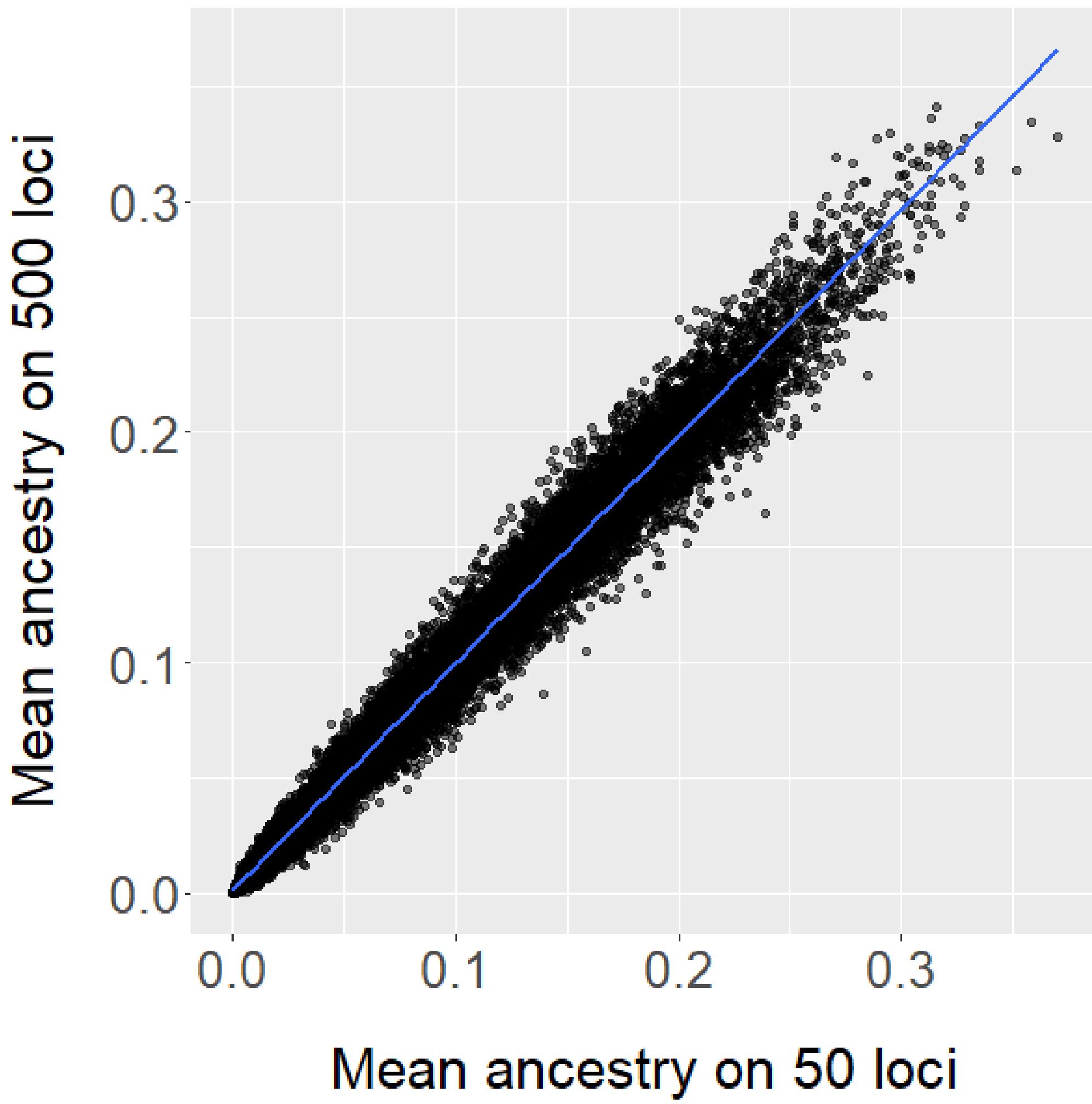

Supplementary Figure S4. Relationship between the mean Neanderthal ancestry estimated from simulations of 50 and 500 independent neutral loci. A total of 20,000 paired simulations were performed using the same parameter combinations and dataset as in the main analysis. For each parameter combination, one simulation was run with 50 loci and another with 500 loci, and the resulting mean Neanderthal ancestry across all sampled individuals was compared. Each point represents a single parameter combination. Mean ancestry estimates obtained with 50 loci closely matched those obtained with 500 loci, showing an almost perfect linear correlation (Pearson's correlation = 0.99, p-value = $< 10^{-15}$) and no significant difference between the two sets of simulations (paired t-test, p-value = 0.67). Simulating 500 loci increased computation time by approximately 8.3-fold relative to 50 loci, indicating that 50 loci provide a suitable compromise between estimation accuracy and computational efficiency.

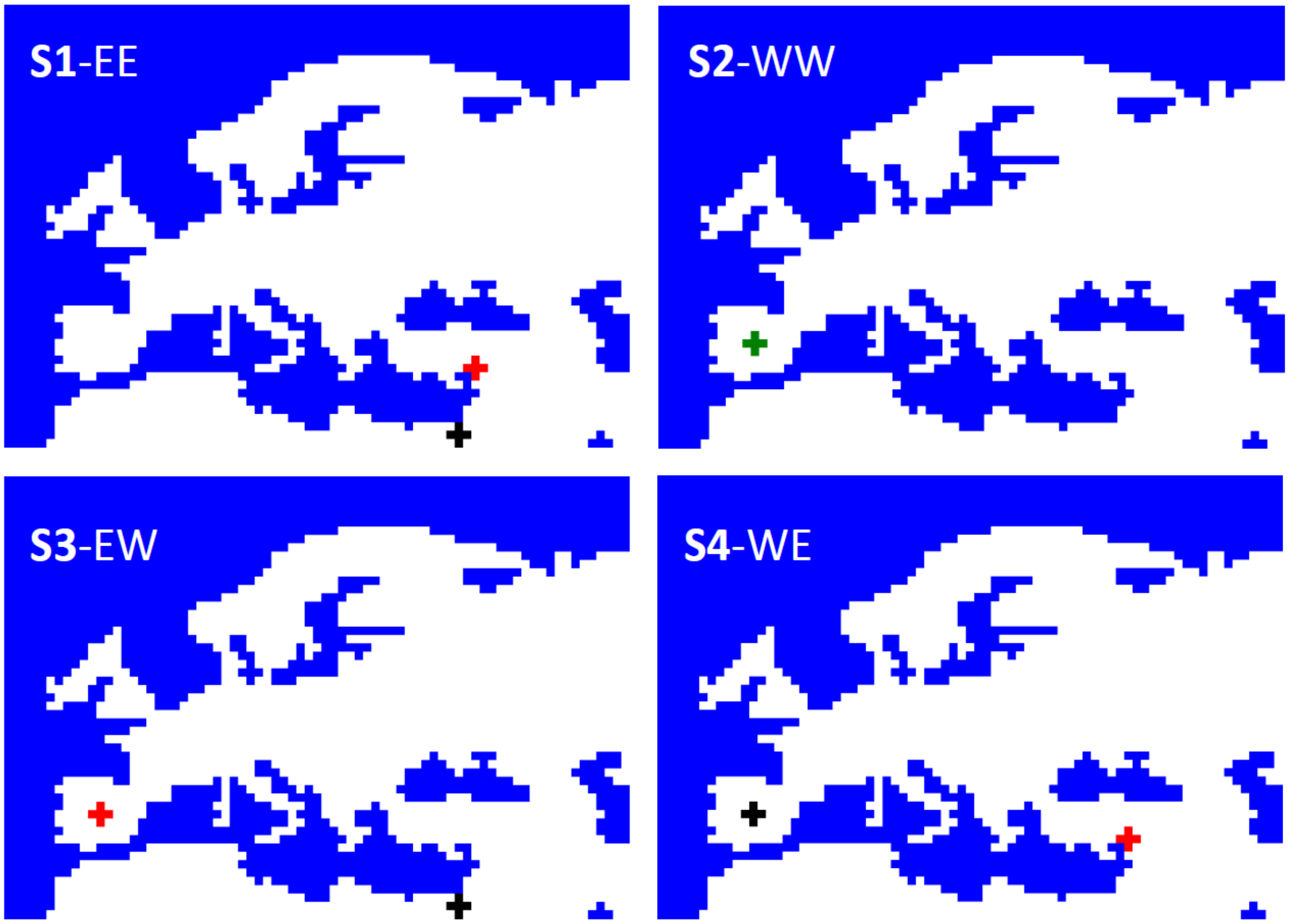

Supplementary Figure S5. The four tested expansion scenarios, defined by the starting locations of HG and Farmers. Black crosses mark HG demes used as origins, red crosses Neolithic farmer demes used as origins, and green crosses demes used as a shared origin. Scenario names are indicated in each panel. The four maps were generated using the SPLATCHE3 program, freely available at www.splatche.com.

Supplementary Table S1. Marginal density *p*-values for the simulated statistics of the four scenarios investigating the Neanderthal habitat latitudinal limits. Estimated on 400,000 simulations per scenario with three tolerance levels, retaining 2,000, 4,000 or 12,000 simulations. Computed with ABCtoolbox2 (*82*).

Tolerance level	Marginal posterior density *p*-value of scenario lat50	Marginal posterior density *p*-value of scenario lat55	Marginal posterior density *p*-value of scenario lat60	Marginal posterior density *p*-value of scenario lat65
0.005	0.689	0.614	0.364	0.280
0.01	0.671	0.523	0.273	0.234
0.03	0.749	0.568	0.324	0.231

Supplementary Table S2. Model choice between the four investigated latitudinal limits of Neanderthal habitat. Performed with abcrf R package (ABC-random forest approach; *83*), using 2,000 trees and 400,000 simulations per scenario. The votes refer to the number of trees that selected each scenario as the most probable one.

Scenario lat50	Scenario lat55	Scenario lat60	Scenario lat65	Chosen scenario	Posterior probability of chosen scenario
566	858	387	189	lat55	0.409

Supplementary Table S3. Proportion of simulations of the four investigated scenarios of northern Neanderthal presence, which produced a gradient of Neanderthal ancestry in HG increasing with distance from the expansion source, a gradient of NE ancestry decreasing with time or both these gradients.

Scenario	Proportion of simulations producing spatially increasing NE ancestry gradient in HG	Proportion of simulations producing temporally decreasing NE ancestry gradient in HG	Proportion of simulations producing both spatial and temporal gradients in HG
Lat50	0.73	0.73	0.65
Lat55	0.80	0.66	0.63
Lat60	0.82	0.55	0.54
Lat65	0.82	0.50	0.49

Supplementary Table S4. Mean difference in Neanderthal (NE) ancestry between hunter-gatherers (HG) and farmers (NF) across simulated scenarios with different expansion origins. For each simulation, the difference was calculated as mean Neanderthal ancestry in HG minus that in NF, and results were then averaged across all simulations of each scenario.

Scenario	Mean NE ancestry difference between HG and NF	Welch's *t*-test *p*-value
S1-EE	0.0065	$<10^{-07}$
S2-WW	0.0258	$<10^{-07}$
S3-EW	-0.0214	$<10^{-07}$
S4-WE	-0.0087	$<10^{-07}$

Supplementary Table S5. Characteristics of the posterior distributions of the estimated parameters and indices of accuracy of the parameter estimation. The estimation was performed on 200,000 simulations of the 3-layered approach, with three tolerance levels, retaining 1,000, 2,000 or 6,000 simulations. Computed with ABCtoolbox2 (*82*). NE stands for Neanderthals, HG for pre-Neolithic hunter-gatherers and NF for Neolithic farmers.

Parameter	Tolerance level	Posterior mean	Posterior mode	Posterior mode relative bias	Mode Root Mean Square Error (RMSE)	Mode Factor2	Mode Pearson's *r* (*p*-value)	Posterior lower HDI95	Posterior upper HDI95
Hybridization rate between NE and HG (γ_H)	0.005	0.006	0.006	0.087	0.000	1.000	0.836 ($<10^{-15}$)	0.004	0.008
	**0.01**	**0.006**	**0.006**	**0.088**	**0.001**	**1.000**	**0.826 ($<10^{-15}$)**	**0.004**	**0.008**
	0.03	0.006	0.006	0.105	0.001	1.000	0.838 ($<10^{-15}$)	0.004	0.008
Assimilation rate of HG into NF populations (γ_A)	0.005	0.091	0.044	1.515	0.059	0.705	0.253 ($<10^{-15}$)	0.001	0.179
	**0.01**	**0.092**	**0.049**	**1.931**	**0.061**	**0.684**	**0.230 ($<10^{-15}$)**	**0.002**	**0.181**
	0.03	0.086	0.042	2.448	0.060	0.677	0.306 ($<10^{-15}$)	0	0.176
Ratio of HG to NE effective population size ($Kratio_{HN}$)	0.005	6.094	6.444	0.442	2.370	0.855	0.048 (0.167)	2.387	9.798
	**0.01**	**6.088**	**7.657**	**0.484**	**2.519**	**0.830**	**0.037 (0.130)**	**2.315**	**9.717**
	0.03	6.000	7.495	0.787	3.084	0.708	0.084 ($<10^{-4}$)	2.283	9.670
Ration of NF to HG effective population size ($Kratio_{FH}$)	0.005	4.924	3.849	0.230	1.401	1.000	0.016 (0.646)	3.101	6.779
	**0.01**	**4.943**	**4.253**	**0.221**	**1.397**	**0.999**	**-0.014 (0.574)**	**3.135**	**6.818**
	0.03	5.002	4.535	0.216	1.357	1.000	0.031 (0.165)	3.183	6.859
Proportion of Long Distance Dispersals (P_{LDD})	0.005	0.003	0.002	1.890	0.002	0.451	-0.024 (0.477)	0	0.008
	**0.01**	**0.003**	**0.002**	**1.813**	**0.002**	**0.481**	**0.021 (0.381)**	**0**	**0.008**
	0.03	0.003	0.002	1.823	0.002	0.475	0.041 (0.061)	0	0.008

Data S1. (separate file)

Information of the genomic data used in the present study. The information provided are: the name assigned to the individual (AADR_VersionID); the study which produced the genome (Publication); if the individual was classified as being a pre-Neolithic hunter-gatherer (HG) or a Neolithic farmer (NF) (Population_Group); the latitude of the area where the individual was found (Latitude); the longitude of the area where the individual was found (Longitude); the age of the individual in years before present (Mean_Age_YBP); the generation at which the individual is sampled dusting the simulations (Simulated_generation); the population sample to which the individual was assigned for estimating the mean sample Neanderthal ancestry and for performing the simulations (Sample); the estimated Neanderthal ancestry of the individual.

Data S2. (separate file)

Information of the population samples into which the individuals were grouped. The information provided are: the name of the sample (Sample_name); if the sample was sampled from the layer of hunter-gatherers (HG) or farmers (NF) (Population_Group); the latitude of the deme where the sample was simulated (Latitude); the longitude of the deme where the sample was simulated (Longitude); the distance of the sample deme form the respective expansion source in *Km* (Distance_from_expansion_source_in_km); the mean age of the individuals in the sample, in years before present (Mean_sample_age_YBP); the generation at which the sample is sampled during the simulations (Simulated_generation); the population sample to which the individual was assigned for estimating the mean sample Neanderthal ancestry and for performing the simulations (Sample); the for NF samples, the sub-division of the Neolithic to which the sample was assigned for linear mixed model analysis (Simulated_period); the mean estimated Neanderthal ancestry of the individuals in the sample (Mean_Neanderthal_ancestry).
